# Malignant phyllodes tumor of the breast with rapid progression: a case report

**DOI:** 10.1186/s40792-020-00986-8

**Published:** 2020-12-07

**Authors:** Hajime Abe, Atsuko Teramoto, Yumiko Takei, Yoshihito Tanaka, Genichiro Yoneda

**Affiliations:** 1 Breast Center, Bell Land General Hospital, 500-3 Higashiyama, Naka-ku, Sakai, Osaka 599-8247 Japan; 2 Department of Breast Surgery, Kansai Medical University Kori Hospital, 8-45, Kori-Hondori Machi, Neyagawa, Osaka 572-8551 Japan; 3 Department of Pathology, Bell Land General Hospital, 500-3 Higashiyama, Naka-ku, Sakai, Osaka 599-8247 Japan

**Keywords:** Malignant phyllodes tumor, Breast, Mastectomy, Chemotherapy, Doxorubicin–ifosfamide

## Abstract

**Background:**

Malignant phyllodes tumors (PTs) of the breast occur infrequently and are difficult to treat with adjuvant therapy. Here, we present a case of a female patient with a huge malignant PT with rapid progression in a short period.

**Case presentation:**

A 44-year-old woman presented to our hospital with a rapid growth mass in her right breast, measuring 20 cm. She was initially diagnosed as having a borderline phyllodes tumor by core needle biopsy and underwent total mastectomy and artificial dermis was grafted, 20 days later, latissimus dorsi muscle flap and free skin grafting were performed. Two courses of doxorubicin–ifosfamide therapy were administered because of recurrence, but the patient died 4 months after the mastectomy.

**Conclusions:**

A standard therapeutic strategy for malignant PTs is needed in urgently to reduce the risk of tumor recurrence.

## Background

Phyllodes tumors (PTs) of the breast are extremely rare, globally accounting for 0.3% to 1% of breast tumors [1]. Their name is derived from the Greek *phyllon* (leaf) because of its lobed histological appearance. It is also known as cystosarcoma phyllodes, adenomatous myxoma, and pseudosarcoma adenoma [1–3]. It usually occurs in middle-aged women (age, 35–55 years) [4]. Clinically, the size of the tumor varies between 4 and 7 cm on average [5], but about one-fifth of PTs are called giant PT tumors because of their uncommon diameter of more than 10 cm [6]. Depending on the histopathological features, PTs are categorized into three grades with different proportions, benign (60%–75%), borderline (13%–26%), and malignant (10%–20%) [7]. Patients suffering from PTs have no specific clinical manifestations, and it is difficult to distinguish the benign subgroup from the borderline and malignant subgroups. Malignant PTs are more readily characterized by stromal pleomorphism and overgrowth, frequent mitoses and infiltrative borders [8]. Lymph node metastasis is rare, and the metastatic path relies mainly on the blood. Surgical removal is the primary treatment for PT, given that adjuvant treatments play a poorly efficient role in this malignancy. Here, we report the case of a female patient with a huge malignant PT with rapid progression in a short period.

### Case presentation

**Fig. 1:**
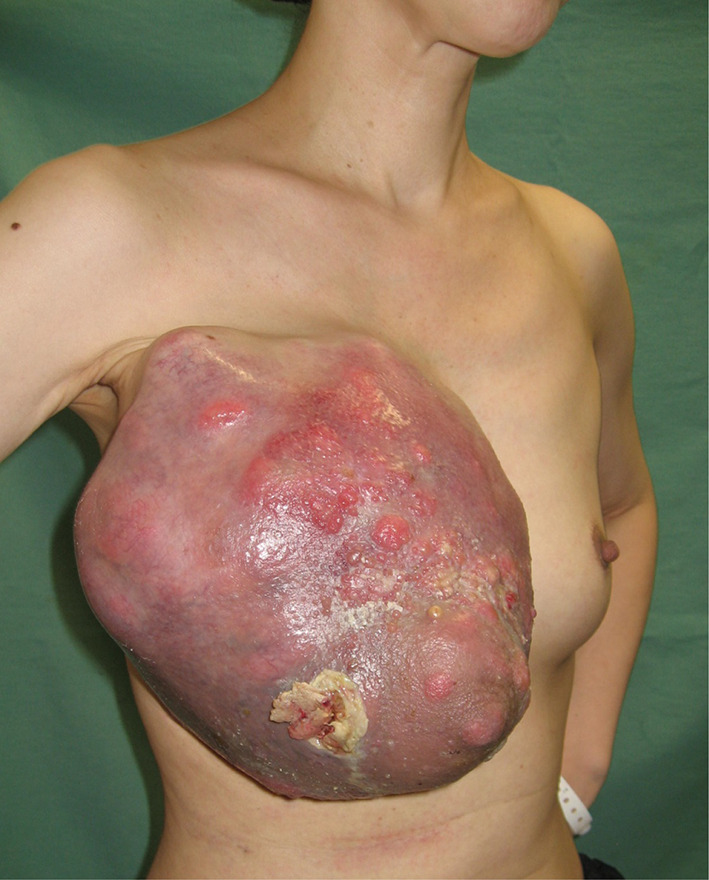
Huge phyllodes tumor with ulceration in the right breast

**Fig. 2:**
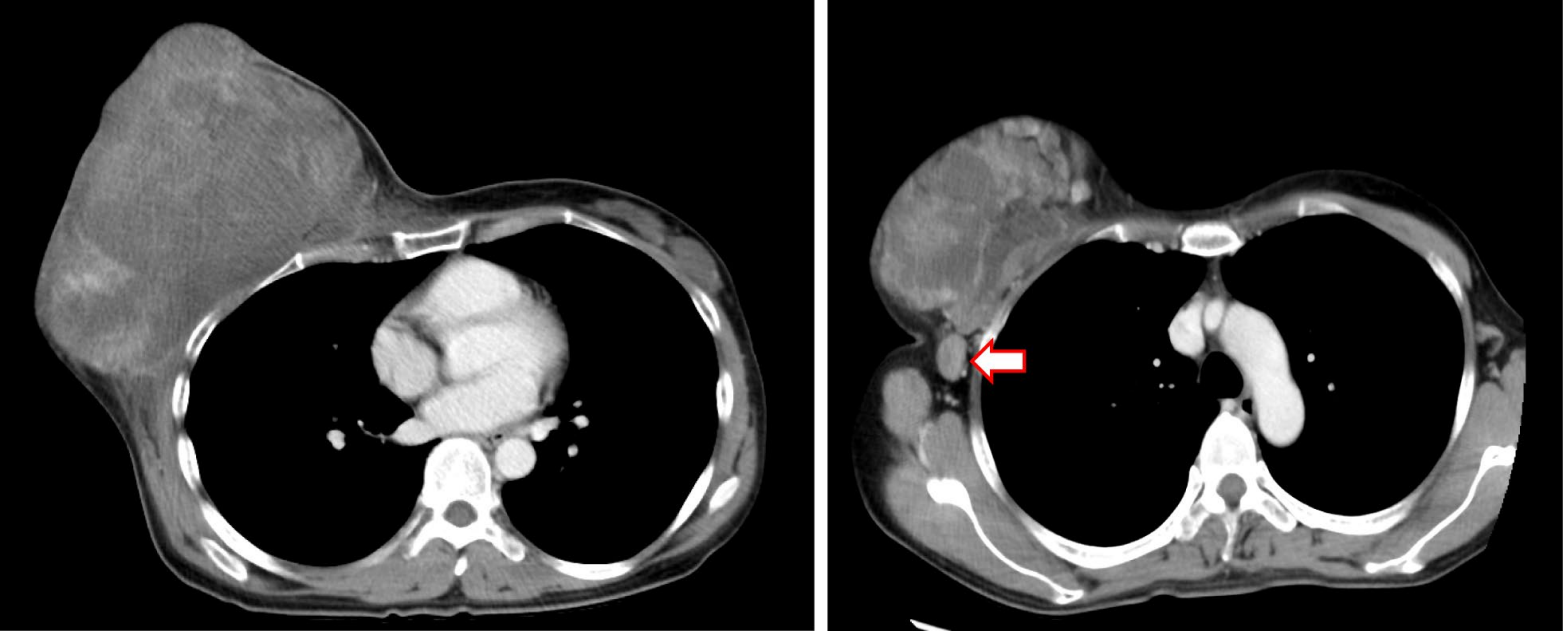
Computed tomography showed a 20 cm heterogeneous mass in the right breast and axillary and supraclavicular lymph nodes swelling (arrow)

**Fig. 3:**
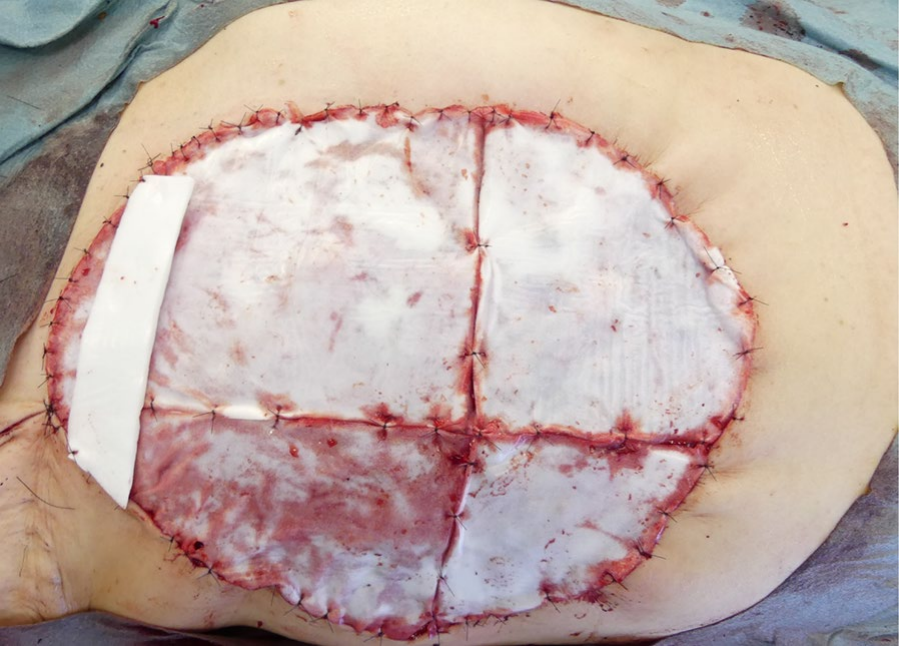
Right mastectomy with axillary lymph node dissection covered with artificial skin

**Fig. 4:**
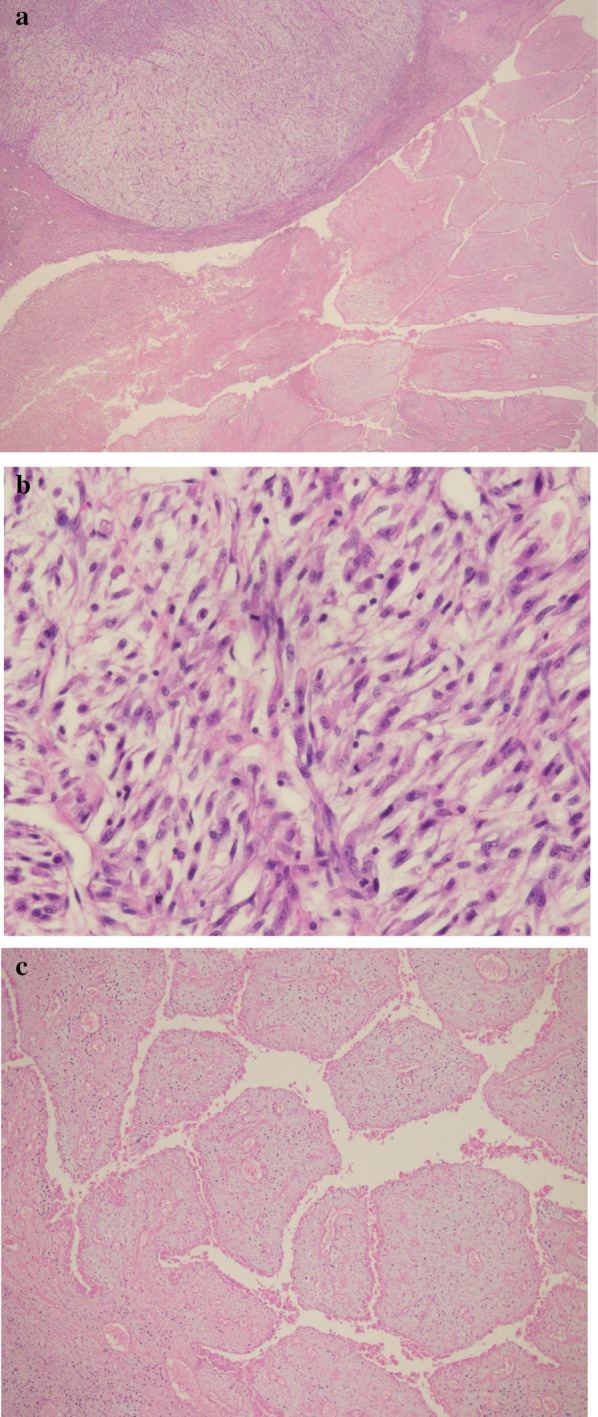
The histopathological findings revealed atypical spindle-shaped cells with enlarged nuclei and exhibited a fibrosarcoma-like morphology (**a**) (HE stain, × 2). Numerous mitoses were noted (**b**) (HE stain, × 40). A degenerated leaf-like structure was found in the center of the lesion (**c**) (HE stain, × 10)

A 44-year-old woman presented to our hospital with a rapid growth mass in her right breast, measuring 20 cm. The breast skin appeared dark with ulceration (Fig. 1). Her family history and past history were unremarkable. One year prior to admission, she incidentally palpated the tumor, and noted gradual growth 2 months prior to the hospital visit. Computed tomography and magnetic resonance imaging revealed a hemorrhagic giant mass with a well-defined border. The axillary and supraclavicular lymph nodes were swollen, but there was no sign of chest wall invasion or distant metastasis (Fig. 2). A diagnosis of PT of borderline malignancy was established on the basis of the core needle biopsy findings. Subsequently, the patient underwent mastectomy of the right breast and axillary lymph node dissection. During surgery, the surgeons detected invasion of the pectoralis major; thus, the partial muscle adhering to the tumor was resected. Because the skin defect was large, artificial dermis [9] was grafted to remedy for the defect (Fig. 3). Twenty days later, latissimus dorsi muscle flap and free skin grafting were performed. The histopathological examination demonstrated that the tumor was composed of atypical spindle-shaped cells with enlarged nuclei and exhibited a fibrosarcoma-like morphology which included ill-defined invasion into the adjacent breast tissue, the overlying skin, and the pectoral muscle (Fig. 4a). Numerous mitoses were noted (10/10 high-power fields) and necrosis had occurred (Fig. 4b). A degenerated leaf-like structure was observed in the center of the lesion (Fig. 4c). Three lymph node metastases with a maximum diameter of 25 mm. were noted. Based on these findings, a final diagnosis of malignant PT was established.

**Fig. 5:**
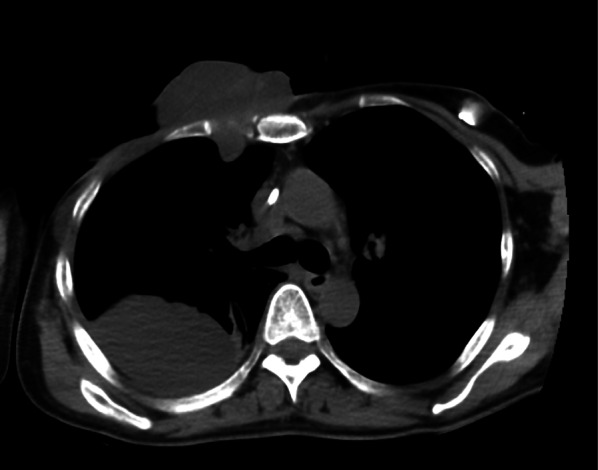
Computed tomography revealed regrowth of tumor in the right chest, right lung and right pleural effusion

**Fig. 6:**
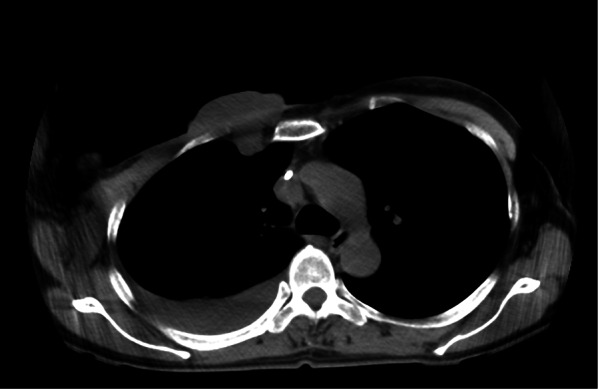
Computed tomography showed a mild reduction in chest wall tumor and pleural dissemination after one cycle of AI therapy

**Fig. 7:**
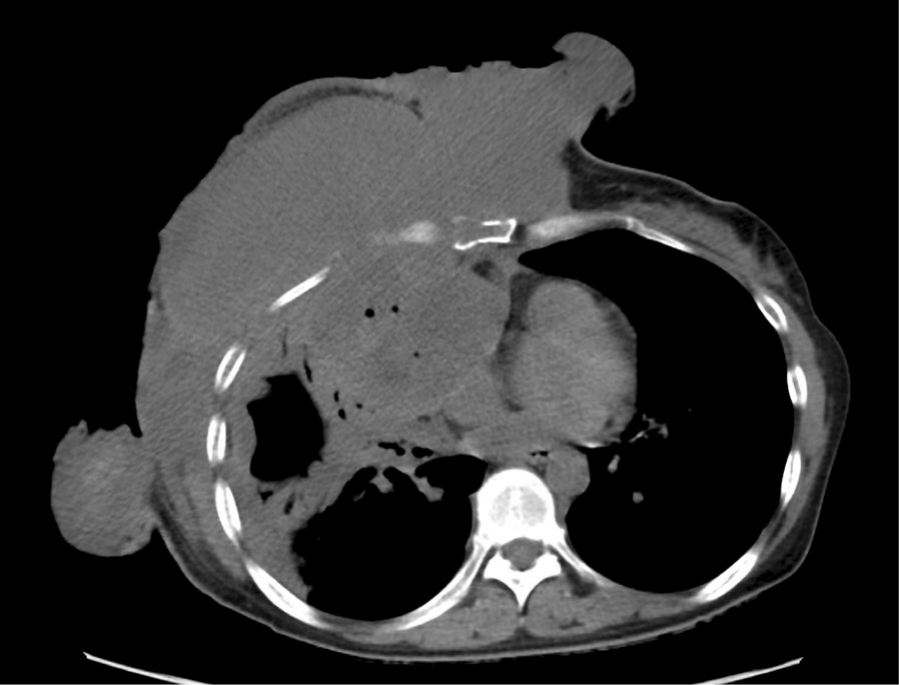
Computed tomography showed a rapid increase in right chest wall tumors and pleural dissemination

Because 1 month after the mastectomy the tumor had re-grown in the surrounding skin graft and right pleural effusion had appeared (Fig. 5), as an alternative treatment, we administered two courses of doxorubicin–ifosfamide (AI) therapy (30 mg/m^2^ doxorubicin on days 1–2 and 2 g/m^2^ ifosfamide on days 1–5) including Mesna (sodium 2-mercaptoethane sulfonate) and sufficient infusion volumes to prevent ifosfamide-related hemorrhagic cystitis. Grade 4 neutropenia and anemia (as defined by the Common Terminology Criteria for Adverse Events) occurred during AI therapy, thus, to prevent the advancement of neutropenia, filgrastim, a granulocyte-colony-stimulating factor, was administered, and transfusion was performed. The local tumor was temporarily reduced after one cycle of AI therapy (Fig. 6); however, after two cycles of AI therapy, the chest wall recurrence and pleural dissemination progressed rapidly (Fig. 7). The patient died 4 months after the mastectomy because of respiratory failure.

## Discussion

Malignant PT is rare lesion of the breast that can mimic benign masses such as fibroadenomas, on clinical diagnosis, but is characterized by a typical rapid growth. PTs usually occur in middle-aged women ranging in age from 35 to 55 years, with an average presentation at 45 years [4]. PTs are composed of epithelial elements and a connective tissue stroma with higher stromal cellularity. A malignant PT is distinguished from a benign/borderline PT by the presence of marked stromal cellularity, cellular atypia and mitotic activity in at least 10/10 high-power fields [10].

The clinical presentation and the radiographic findings of malignant PT are strikingly similar to those of benign lesions, such as fibroadenoma, or even benign PT, thus, making it quite challenging for clinicians to diagnose or even to suspect the disease at an early stage. Although routine breast biopsy may not be warranted, it is crucial for clinicians to consider and include PT in their differential diagnosis. Moreover, it is also evident that clinicians cannot rely completely on radiographic findings.

According to the National Comprehensive Cancer Network (NCCN) guidelines for breast cancer, the management of PTs with a size > 3.0 cm is surgical excision with clean margins (≥ 1.0 cm) without axillary staging, regardless of whether the tumor is benign, borderline, or malignant [11]. Many other studies have supported the contention that margins that are ≤ 1.0 cm are associated with a higher recurrence rate, ranging from 16.7 to 40% [4, 12].

The prognosis of PTs is variable, with local recurrence rates ranging from 10 to 40% (average 15%) and distant metastases occurring in 10% of all PTs and up to 20% of malignant PTs [13]. Survival after metastatic disease is poor, with various case series reporting a median survival ranging from 4 to 17 months, with large variability based on the site of the metastatic disease [14]. Other large prospective studies have reported 5-year disease-free survival rates of 96% for benign PTs and 66% for malignant PTs [15]. Most sarcomas metastasize hematogenously, and the incidence of axillary lymph node involvement in malignant PTs ranges from 1.1 to 3.8% [16].

There is currently no consensus regarding the recommendations for radiotherapy, hormonal therapy, and systemic chemotherapy for malignant PTs. To date, no double-blinded, multicenter study has been performed on this subject. Most case reports and studies describe treating these tumors exclusively with wide local excision, in according with the current NCCN guidelines. As PTs are considered as soft-tissue sarcoma, adjuvant chemotherapy with doxorubicin plus dacarbazine may provide some benefits to patients with large (> 5.0 cm), high-risk tumors [17]. A deeper investigation of the addition of adjuvant therapy for large aggressive malignant cases of PT may prove to be fruitful [18]. Recently, doxorubicin and ifosfamide therapy has been reported to be effective to treat metastases of malignant PTs [19, 20]. In accordance with previous reports [19, 20], we administered AI therapy using 60 mg/m^2^ doxorubicin and 10 g/m^2^ ifosfamide in each course.

Because there are not many reports of malignant cases of PT, the data available are insufficient to calculate fully the statistics of the survival rate associated with these tumors. Past case reports and studies have suggested that the prognosis of malignant PT of the breast is usually poor, while the overall prognosis of benign PT is good [14, 21].

The early diagnosis and staging of PTs are pivotal not only for improving the overall outcome of the disease after treatment, but also to promote the quality of life of the patient by causing less disfiguration. While the breast cancer screening guideline suggests that women over 40 years of age should begin routine mammograms to detect the presence of breast cancers, PTs can occur a decade before this minimum screening age as they occur during the third or fourth decades of life. Moreover, if the patient suspects that the lesion exhibits growth within 6 months to a year of initial detection, it should be considered for further workup.

## Conclusions

In conclusion, malignant PTs are rare entities with distinct clinicopathological features. These tumors should be accurately recognized and effectively treated at first diagnosis, as they have a high risk of recurrence. There is no established consensus regarding the optimal type of surgery and indications for radiotherapy and chemotherapy regimens in these cases. The establishment of standard therapeutic strategy for malignant PTs is needed urgently to reduce the risk of tumor recurrence.

## Data Availability

All datasets supporting the conclusions of this article are included within the article.

## Abbreviations

AI: Doxorubicin–ifosfamide
NCCN: National Comprehensive Cancer Network
PT: Phyllodes tumor
